# Somaclonal variations and their applications in horticultural crops improvement

**DOI:** 10.1007/s13205-016-0389-7

**Published:** 2016-02-13

**Authors:** Hare Krishna, Mahdi Alizadeh, Dhurendra Singh, Udayvir Singh, Nitesh Chauhan, Maliheh Eftekhari, Radha Kishan Sadh

**Affiliations:** 1 ICAR-Central Institute for Arid Horticulture, Beechwal, Bikaner, Rajasthan 334 006 India; 2 Department of Horticulture, Faculty of Agriculture, Gorgan University of Agricultural Sciences and Natural Resources (GUASNR), Golestan, Gorgan, Iran

**Keywords:** Micropropagation, Somaclones, Oxidative stress, Epignetic variation, Molecular markers, Crop improvement

## Abstract

The advancements made in tissue culture techniques has made it possible to regenerate various horticultural species in vitro as micropropagation protocols for commercial scale multiplication are available for a wide range of crops. Clonal propagation and preservation of elite genotypes, selected for their superior characteristics, require high degree of genetic uniformity amongst the regenerated plants. However, plant tissue culture may generate genetic variability, i.e., somaclonal variations as a result of gene mutation or changes in epigenetic marks. The occurrence of subtle somaclonal variation is a drawback for both in vitro cloning as well as germplasm preservation. Therefore, it is of immense significance to assure the genetic uniformity of in vitro raised plants at an early stage. Several strategies have been followed to ascertain the genetic fidelity of the in vitro raised progenies comprising morpho-physiological, biochemical, cytological and DNA-based molecular markers approaches. Somaclonal variation can pose a serious problem in any micropropagation program, where it is highly desirable to produce true-to-type plant material. On the other hand, somaclonal variation has provided a new and alternative tool to the breeders for obtaining genetic variability relatively rapidly and without sophisticated technology in horticultural crops, which are either difficult to breed or have narrow genetic base. In the present paper, sources of variations induced during tissue culture cycle and strategies to ascertain and confirm genetic fidelity in a variety of in vitro raised plantlets and potential application of variants in horticultural crop improvement are reviewed.

## Introduction

Plant tissue culture techniques proffer a substitute method of vegetative propagation of horticultural crops (Krishna et al. 2005; Alizadeh et al. 2010). Clonal propagation through tissue culture (popularly known as micropropagation) can be realized relatively rapidly within a small space (Krishna et al. 2008; Eftekhari et al. 2012). The uniformity of individual plants within a clone population is a major advantage of clonal cultivars in commercial production (Krishna and Singh 2013). However, genetic variations do occur in undifferentiated cells, isolated protoplasts, calli, tissues and morphological traits of in vitro raised plants (Bairu et al. 2011; Currais et al. 2013). In 1981, Larkin and Scowkraft coined a general term “somaclonal variation” for plant variants derived from any form of cell or tissue cultures.

At present, micropropagated plants, in various crops, such as strawberry, papaya, banana, grapes, pineapple, citrus, tomato, cucumber, watermelon, rhododendron, orchids, etc., are preferred over plants propagated through conventional means. However, ever since the first formal report of morphological variants in sugarcane plants produced in vitro in 1971 (Heinze and Mee 1971), several instances of somaclonal variations have been reported in different horticultural crops. The notable example could be banana in which occurrence of off-types from tissue cultured plantlets ranged from 6 to 38 % in Cavendish cultivars (Sahijram et al. 2003); however, it could be as high as 90 % (Smith 1988). From the point of commercial micropropagation, variation of any kind, in particular, genetic variations may be considered obstructive and worthless; since, such variations may lead to loss of genetic fidelity. However, plant cell and tissue cultures render increased genetic variability comparatively faster and without applying a sophisticated technology. This technology holds ample scope in crop improvement of horticultural crops, which are largely propagated vegetatively, partly, due to reasons like longer juvenile phase as in perennial fruit crops, occasional inbreeding depression, self and cross incompatibility, narrow genetic base especially in ornamentals, etc. Further, somaclonal variations require less space and time for screening of desirable traits in vitro unlike cross seedlings of perennial crops, which require a great deal of land area and time. Somaclones may itself have numerous applications in plant breeding and genetic improvements and recovery of such novel variants can be enhanced by applying suitable in vitro selection pressure (Jain 2001; Lestari 2006).

## Sources of variations detected in plant tissue culture

Tissue culture is an efficient method of clonal propagation; however, the resulting regenerants often has a number of somaclonal variations (Larkin and Scowcroft 1981). These somaclonal variations are mainly caused by newly generated mutations arising from tissue culture process (Sato et al. 2011b). The triggers of mutations in tissue culture had been attributed to numerous stress factors, including wounding, exposure to sterilants during sterilization, tissue being incomplete (protoplasts as an extreme example), imbalances of media components such as high concentration of plant growth regulators (auxin and cytokinins), sugar from the nutrient medium as a replacement of photosynthesis in the leaves, lighting conditions, the disturbed relationship between high humidity and transpiration (Joyce et al. 2003; Sato et al. 2011b; Smulders and de Klerk 2011).

**Fig. 1:**
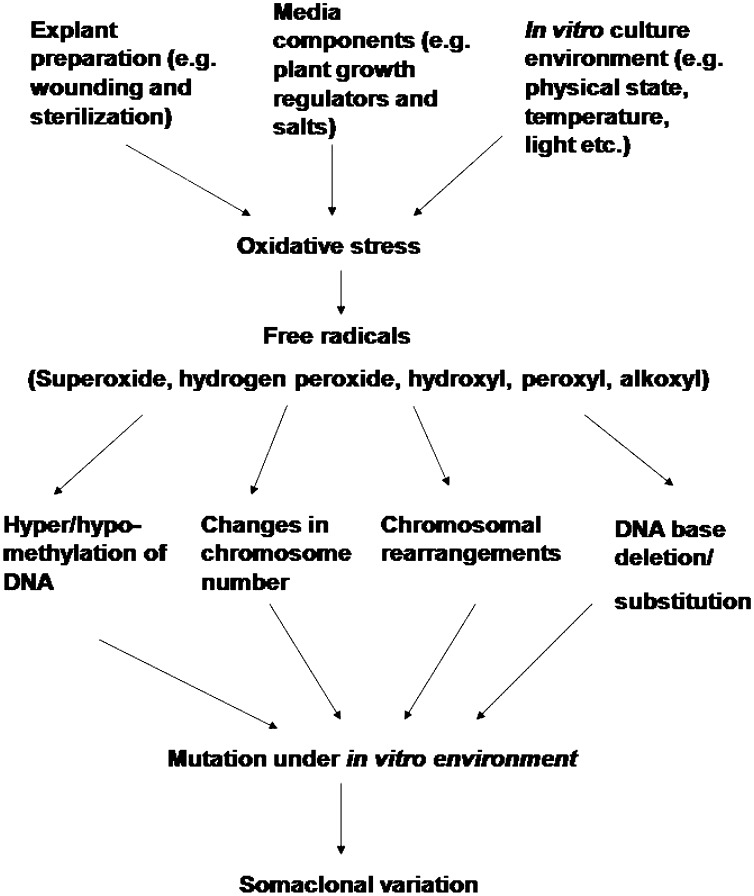
Mechanism of somaclonal variation in micropropagated plants as a result of oxidative burst upon in vitro culture

Much of the variability expressed in micropropagated plants may be the result of, or related to, oxidative stress damage inflicted upon plant tissues during in vitro culture (Cassells and Curry 2001; Tanurdzic et al. 2008; Nivas and DSouza 2014). Oxidative stress results in elevated levels of pro-oxidants or reactive oxygen species (ROS) such as superoxide, hydrogen peroxide, hydroxyl, peroxyl and alkoxyl radicals. These ROS may involve in altered hyper- and hypo-methylation of DNA (Wacksman 1997); changes in chromosome number from polyploidy to aneuploidy, chromosome strand breakage, chromosome rearrangements, and DNA base deletions and substitutions (Czene and Harms-Ringdahl 1995), which in turn may lead to mutations in plant cells in vitro (Fig. 1). Somaclonal variation shows a similar spectrum of genetic variation to induced mutation as both of them result in qualitatively analogous gamut of DNA changes (Cassells et al. 1998). Different factors affect the frequency of development of somaclones under in vitro conditions.

### Explant/explant source

Differences in both the frequency and nature of somaclonal variation may occur when regeneration is achieved from different tissue sources (Sahijram et al. 2003). Highly differentiated tissues such as roots, leaves, and stems generally produce more variations than explants with pre-existing meristems, such as axillary buds and shoot tips (Duncan 1997). In general, the older and/or the more specialized the tissue is used for regeneration, the greater the chances that variation will be recovered in the regenerated plants (Table 1) as under such conditions, adventitious shoot regeneration (shoot organogenesis) takes place from atypical points of origin directly or indirectly through a callus stage (e.g., from leaves, petioles, shoot internodes, root segments, anthers, hypocotyls, cotyledons, etc.; Pijut et al. 2012). Somaclonal variation can also arise from somatic mutations already present in the donor plant, i.e., presence of chimera in explants (Karp 1994).

**Table 1:** Occurrence of somaclonal variations as affected by the choice of explants

S. no.	Crop species	Explants/explants source	Presence or absence of somaclonal variations (+/−)	References
1	African violet (*Saintpaulia* sp.)	Leaf segments	+	Matsuda et al. (2014)
2	Almond (*Prunus dulcis*)	Axillary branching	−	Martins et al. (2004)
3	Chimeric ‘Maricongo’ banana	Vegetative and floral axis tip	+	Krikorian et al. (1993)
	Cavendish group of bananas (*Musa* sp.)	Chimeric shoot tip	+	Israeli et al. (1995)
	Banana cv. Martaman	Shoot tip	−	Ray et al. (2006)
4	Brinjal (*Solanum melongena*)	Hypocotyl	−	Mallaya and Ravishankar (2013)
		Callus induction on leaves, nodes and intermodal explants	+	Naseer and Mahmood (2014)
5	Chrysanthemum (*Dendranthema grandiflora*)	Callus from leaves and internodes	+	Miler and Zalewska (2014)
6	European violet (*Viola uliginosa* Besser)	Leaf and petiole fragments	+	Slazak et al. (2015)
7	Gerbera (*Gerbera* *jamesonii* Bolus)	Capitulum	−	Bhatia et al. (2009, 2011)
8	Gloxinia	Leaf explants	+	Hu and Xu (2010)
9	*Hedychium coronarium* Koen.	Axillary bud explants	−	Parida et al. (2013)
10	Hop (*Humulus lupulus* L.)	Meristem tissue	−	Patzak (2003)
11	*Kaempferia galanga*	Buds of rhizomes	−	Mohanty et al. (2011)
12	Kiwifruit (*Actinidia deliciosa* (Chev.) Liang and Ferguson) cv. ‘Tomuri’	Leaf blades and petioles	+	Prado et al. (2007)
13	Oil palm (*Elaeis guineensis* Jacq.)	Mature zygotic embryos	+	Rival et al. (2013)
		Immature zygotic embryo	+	Sanputawong and Te-chato (2011)
		Immature leaves	+	Lucia et al. (2011)
14	Papaya (*Carica papaya* L.)	Axillary shoot tips underwent cryopreservation	+	Kaity et al. (2009)
15	Patchouli (*Pogostemon patchouli*)	Callus induction on internodal and leaf explants	+	Ravindra et al. (2012)
16	Potato (*Solanum tuberosum*)	Callus cultures of stem explant	+	Thieme and Griess (2005)
		Callus induction via fresh sprouts	+	Munir et al. (2011)
17	Sweet cherry (*Prunus avium*)	Shoot apical portions	+	Piagnani and Chiozzotto (2010)
18	Rootstock Mr.S 2/5, selected from a half-sib progeny from *Prunus cerasifera* Erhr	Leaf	+	Muleo et al. (2006)
19	*Swertia chirayita*	Axillary multiplication	−	Joshi and Dhawan (2007)
20	Turmeric (*Curcuma longa* L.)	Latent axillary buds of rhizome	−	Nayak et al. (2010)
		Axillary buds of unsprouted rhizome	−	Panda et al. (2007)
		Callus cultures established from rhizome segments	+	Kar et al. (2014)
21	*Vitis* spp.	Nodal segment	−	Alizadeh et al. (2008)

### Mode of regeneration

Both culture initiation and subsequent subculture expose explants to oxidative stress (Krishna et al. 2008), which may result in mutations (Cassells and Curry 2001). It seems evident that ‘extreme’ procedures such as protoplast culture and also callus formation impose stress (Smulders and de Klerk 2011). Magnitude of this stress depends on the tissue culture technique. Therefore, the production of plants via axillary branching does not normally result in the production of variants, while cultures that go through a callus phase are the ones that theoretically promote a higher mutation rate (Zayova et al. 2010).

Investigations indicate more chromosome variability in the callus phase than in adventitious shoots (Saravanan et al. 2011), indicating a loss of competence in the more seriously disturbed genomes. This could be explained by the different grade of disturbance with which the cells are confronted. In the first case, cells follow a pattern of division which is the normal one in the developing plant. On the other hand, callus formation implies a dedifferentiation phase followed by uncontrolled cell divisions (Vázquez 2001). Some types of tissue culture mimic, in some aspects, other stressful situations as, for example, protoplast preparation in which cell wall degradation resembles the infective process of some pathogens. Therefore, the type and magnitude of the stress imposed on cultured cells varies according to the technique used. In contrast to popular belief that the growth of unorganized callus is necessary for induction of genetic variation, variability could be noticed in plants regenerated from explants adventitiously (Farahani et al. 2011; Bhojwani and Dantu 2013).

Sometimes for regeneration under in vitro conditions, somatic embryogenesis is the preferred pathway for generating propagules. It has been suggested that regeneration via embryogenesis has better chance of obtaining genetically uniform plants than through organogenic differentiation (Vázquez 2001). This is so, because DNA in the initial stages of development in somatic embryogenesis contains lower levels of methylation than in the later stages (Sahijram et al. 2003). Variation in in vitro cultures raised through somatic embryogenesis has been reported in several horticultural crops like hazel nut (Diaz-Sala et al. 1995), *Citrus paradisi* (Hao et al. 2004), oil palm (Jaligot et al. 2004), rose (Xu et al. 2004), potato (Sharma et al. 2007), grapevine (Schellenbaum et al. 2008), coffee (Menéndez-Yuffá et al. 2010), olive (Leva et al. 2012), tamarillo (Currais et al. 2013) and brinjal (Naseer and Mahmood 2014).

### Effect of length of culture period and number of subculture cycles

The longer a culture is maintained in vitro, the greater the somaclonal variation is (Kuznetsova et al. 2006; Gao et al. 2010; Farahani et al. 2011; Jevremović et al. 2012; Sun et al. 2013). Variant karyotypes are found to amass with increasing age of callus and as a result the chances of variant plants produced during successive subculture also increases, in general (Zayova et al. 2010). Furthermore, the rapid multiplication of a tissue, during micropropagation, may affect its genetic stability. Khan et al. (2011) reported that after the eighth subculture, the number of somaclonal variants increased with a simultaneous decrease in the multiplication rate of propagules in banana.

Similarly, Clarindo et al. (2012) suggested a limit of less than 4 months storage of coffee cell aggregate suspensions for true-to-type mass propagation as ploidy instability was noticed in long-term in vitro culture. Similarly when Farahani et al. (2011) raised olive cultivars, under in vitro conditions, through internode cuttings, significant difference was observed in morphological characters among the regenerated plants after seventh subculture, which was later confirmed by RAPD analysis. However, C-value analysis showed that no significant change has occurred during subculturing in both olive genotypes. This indicates that the genetic changes accompanied by somaclonal variation could be due to the changes in the nucleotide content of the genome, probably, owing to mutations (insertions/deletions) and not due to quantitative changes.

Not only the number of subculture but their duration also contributes to enhancing the rate of somaclonal variations, especially cell suspension and callus cultures (Bairu et al. 2006; Sun et al. 2013). Studies have shown that somaclonal variation is more apparent in plants regenerated from long-term cultures (Etienne and Bertrand 2003). Rival et al. (2013) noticed that in vitro proliferation induces DNA hypermethylation in a time-dependent fashion and changes in DNA methylation is involved in modulating the expression of embryogenic capacity of oil palm during tissue culture.

### Culture environment

External factors like growth regulators, temperature, light, osmolarity and agitation rate of the culture medium are known to influence the cell cycle in vivo in plants, considerably, which indicates that inadequate control of cell cycle in vitro is one of the causes of somaclonal variation (Karp 1994; Nwauzoma and Jaja 2013). Normal cell cycle controls, which prevent cell division before the completion of DNA replication, are presumed to be disrupted by tissue culture, resulting in chromosomal breakage (Phillips et al. 1994). Chromosome breakage and its consequences (deletions, duplications, inversions, and translocations) cause aberrations in vitro (Duncan 1997). Plant growth regulators can affect the rate of somaclonal variation both directly and indirectly by increasing the multiplication rate and inducing adventitious shoots (Gao et al. 2010). According to D’Amato (1985), it cannot be excluded that some plant growth regulators (PGRs) at certain concentrations or in combination with other growth regulators and/or particular constituents of a culture medium, may act as mutagens.

Several growth regulators, such as 2,4-dichlorophenoxy acetic acid (2,4-D), naphthalene acetic acid (NAA) and BAP (6-benzylaminopurine), synthetic phenylurea derivatives (4-CPPU, PBU and 2,3-MDPU) have been most frequently considered to be responsible for genetic variability (Siragusa et al. 2007; Sun et al. 2013; Sales and Butardo 2014).

Prolonged cultivation in medium containing 2,4-D influences higher DNA ploidy levels in callus cells (da Silva and Carvalho 2014). In their experiment with banana, Sales and Butardo (2014) observed that addition of synthetic auxin 2,4-D in culture medium led to high level of methylation events, particularly, cytosine methylation either at the internal or external cytosine end, which largely resulted in variations in tissue cultured plants. Alteration in genomic DNA methylation rate is being attributed for the development of ‘mantled’ somaclonal variant in oil palm (Eeuwens et al. 2002; Jaligot et al. 2011). Similarly, Arnhold-Schmitt (1993) observed that indole-3-acetic acid (IAA) and inositol in the growth medium induced DNA rearrangements and methylation changes in carrot (*Daucus carota*) callus cultures. Matsuda et al. (2014) observed that percentage of somaclonal variations dramatically increased when PGRs (0.5 ppm BA and 0.1 ppm NAA) were added to the medium inoculated with leaf/leaf segments explants of African violet.

Kinetin has been shown to cause extensive hypomethylation of DNA in proliferating cultures of carrot root explants within 2 weeks (Arnhold-Schmitt 1993), and auxins, including NAA, have the opposite effect and cause hypermethylation (LoSchiavo et al. 1989). Moreover, there is evidence that differential expression in chromatin remodeling genes and histone methylation genes happens during tissue culture, which leads to disruption in the methylation pathway in a non-specific manner and hypo/hypermethylation patterns of DNA induced in tissue culture. This can be stabilized and transmitted to plants regenerated from these cultures (Shearman et al. 2013). Not only the concentration, but also the ratio of different growth regulators affects the occurrence of variations in vitro. Eeuwens et al. (2002) observed that, in general, a relatively high auxin/cytokinin ratio resulted in the lowest incidence of variant ‘mantled’ flowering in oil palm, while using media supplemented with relatively high cytokinins/auxin ratio resulted in a high incidence of mantled flowering. The role of cytokinin was further confirmed by Ooi et al. (2013), who noticed that the mantled inflorescences of oil palm contained higher levels of cytokinins like isopentenyladenine 9-glucoside and lower levels of trans-zeatin 9-glucoside, dihydrozeatin riboside, and dihydrozeatin riboside 5′-monophosphate compared with normal inflorescences.

### Genotype and ploidy

Though, the in vitro morphogenesis seems to be highly dependent on plant growth regulators and media used for culture, it is again genotype specific (Alizadeh et al. 2010; Eftekhari et al. 2012). Among factors affecting somaclonal variation, plant genotype is probably the most important determinant of variation (Shen et al. 2007; Tican et al. 2008; Nwauzoma and Jaja 2013). Earlier, Eeuwens et al. (2002) characterized oil palm clones as low/moderate risk and high risk with regard to ‘mantle’ flowering (wherein anther primordia in both male and female flowers turn into fleshy supplementary carpels), on the basis of terminal inflorescence data generated under in vitro conditions. Clones classified as high risk at the outset gave a significantly higher incidence of mantled flowering in the field than low/medium risk clones, confirming that data on terminal inflorescences produced in vitro allows effective screening of material with regard to the risk of mantled flowering. It is likely that this result from a combination of differences in genotype and differences in epigenetically inherited changes are induced during the pre-embryogenic stages of the culture process, i.e., callus initiation and maintenance.

## Identification of variation in tissue culture

Both genetic and epigenetic alterations are associated with in vitro propagation, which may have phenotypic consequences, and are collectively called somaclonal variation (Larkin and Scowcroft 1981; Guo et al. 2007). As a result, somaclonal variation is characterized by the intricacy of the changes, which are exhibited at various levels, including phenotypic, cytological, biochemical and genetic/epigenetic (Kaeppler et al. 2000). Therefore, the strategy for the detection of somaclones should be based on such manifestations.

A wide variety of tools are available for the detection and characterization of somaclonal variants which are primarily based on the differences in morphological traits (Pérez et al. 2009, 2011; Nhut et al. 2013), cytogenetical analysis for the determination of numerical and structural variation in the chromosomes (Clarindo et al. 2012; Currais et al. 2013; Abreu et al. 2014), biochemical (Vujovic et al. 2010; Kar et al. 2014), molecular DNA markers (Krishna and Singh 2007; Pathak and Dhawan 2012; Hossain et al. 2013; Bello-Bello et al. 2014) or their combinations (Horáček et al. 2013; Dey et al. 2015; Stanišić et al. 2015). The best test for assessing somaclonal variation is to fruit out the plants and conduct an extensive horticultural evaluation, which is unfortunately a long-term endeavor with woody fruit crops, particularly (Grosser et al. 1996). Every tool has its own advantages and limitations in assessment of the variations (Table 2), which govern their use for restricted or large-scale application. The choice of technique for any given application depends upon the material used and the nature of the question being addressed (Karp 2000).

**Table 2:** Strengths and weaknesses of different marker systems for the assessment of clonal fidelity

Advantages	Disadvantages
**Morphological traits**	
Visual differentiation	Sensitive to ontogenic changes and other environmental factors
Does not require any laboratory facility	Limited in numbers
Suitable for preliminary detection	Time-consuming
**Cytological markers (flow-cytometry)**	
Sample preparation and analysis is convenient and rapid in case of in flow-cytometry	Cytosolic compounds may interfere with quantitative DNA staining in flow-cytometry
Rapid and efficient method for routine large-scale studies of ploidy level	Absence of a set of internationally agreed DNA reference standards in case of in flow-cytometry
Unfailing detection of even the smallest modifications in chromosome number	Time-consuming chromosome counting
**Isozyme markers**	
Codominant expression	Sensitive to ontogenic changes and other environmental factors
Ease of performance	Limited in numbers Not all of these reagent systems work efficiently with all plant species Tissue-specific expression
**DNA markers**	
Codominant expression Any source DNA can be used for the analysis Phenotypically neutral Not sensitive to ontogenic changes and other environmental factors Capability to detect culture-induced variation both at the DNA sequence and methylation pattern levels	RAPD markers are dominant and do not permit the scoring of heterozygous individuals. Besides, they exclusively identify sequence changes Possible non-homology of similar sized fragments as ISSR is a multilocus technique Disadvantages of AFLPs include the need for purified, high molecular weight DNA, the dominance of alleles and the possible non-homology of comigrating fragments belonging to different loci Involvement of high development costs in SSR markers if adequate primer sequences for the crop species of interest are unavailable. Further, mutations in the primer annealing sites may result in the occurrence of null alleles (no amplification of the intended PCR product), which may lead to errors in scoring

## Molecular basis of somaclonal variation

How a single plant genotype can result in a variety of phenotypic outcomes under the same in vitro culture conditions is still far from being completely understood. Several bases for somaclonal variation have been proposed, which include changes in chromosome number (Mujib et al. 2007; Leva et al. 2012), point mutations (D’Amato 1985; Ngezahayo et al. 2007), somatic crossing over and sister chromatid exchange (Duncan 1997; Bairu et al. 2011), chromosome breakage and rearrangement (Czene and Harms-Ringdahl 1995; Alvarez et al. 2010), somatic gene rearrangement, DNA amplification (Karp 1995; Tiwari et al. 2013), changes in organelle DNA (Cassells and Curry 2001; Bartoszewski et al. 2007), DNA methylation (Guo et al. 2007; Linacero et al. 2011), epigenetic variation (Kaeppler et al. 2000; Guo et al. 2006; Smulders and de Klerk 2011), histone modifications and RNA interference (Miguel and Marum 2011), segregation of pre-existing chimeral tissue (Brar and Jain 1998; Vázquez 2001; Ravindra et al. 2012; Nwauzoma and Jaja 2013) and insertion or excision of transposable elements (Gupta 1998; Sato et al. 2011b). In particular, transposable elements are one of the causes of genetic rearrangements in in vitro culture (Hirochika et al. 1996; Sato et al. 2011a).

Tissue culture is reported to activate silent transposable elements, resulting in somaclonal variations. Insertions of transposable elements and retrotransposons can function as insertional mutagens of plant genomes, whereas widespread activation may result in a wide gamut of chromosomal rearrangements (Tanurdzic et al. 2008). In turn, these rearrangements can lead to misregulation of genes, aneuploidy and new transposon insertions (Smulders and de Klerk 2011).

However, many aspects of the mechanisms, which result in somaclonal variations, remain undefined. It is therefore, inevitable to explore the genome-wide change through sequencing of whole-genome of the concerned crop. Next-generation sequencing technology has enabled the whole-genome sequencing of individual plants (Miyao et al. 2012). A new generation of sequencing technologies, from Illumina/Solexa, ABI/SOLiD, 454/Roche, and Helicos, has provided unprecedented opportunities for high-throughput functional genomic research (Morozova and Marra 2008; Metzker 2010).

## Somaclonal variations vis-à-vis crop improvement

Genetic variation is an essential component of any conventional crop breeding program. The typical crop improvement cycle takes 10–15 years to complete and includes germplasm manipulations, genotype selection and stabilization, variety testing, variety increase, proprietary protection and crop production stages. Plant tissue culture is an enabling technology from which many novel tools have been developed to assist plant breeders (Karp 1992; Mathur 2013). Tissue culture-induced somaclonal variation is akin to variations induced with chemical and physical mutagens (Jain 2001) and offers an opportunity to uncover natural variability for their potential exploitation in crop improvement.

Like any other technology, in vitro induced somaclonal variation has its own merits and demerits, like the two sides of the same coin.

### Advantages

The advantages comprise: (1) it is cheaper than other methods of genetic manipulation and does not require ‘containment’ procedures. (2) Tissue culture systems are available for more plant species than can be manipulated by somatic hybridization and transformation at the present time. (3) It is not necessary to have identified the genetic basis of the trait, or indeed, in the case of transformation, to have isolated and cloned it. (4) Novel variants have been reported among somaclones, and evidences indicate that both the frequency and distribution of genetic recombination events can be altered by passage though tissue culture. This implies that variation may be generated from different locations of the genome than those, which are accessible to conventional and mutation breeding (Karp 1992). (5) There is no possibility of obtaining chimeric expression if somaclones are raised through cell culture (Evans 1989).

Somaclonal variation has been most successful in crops with limited genetic systems (e.g., apomicts, vegetative reproducers) and/or narrow genetic bases. In ornamental plants, for instance, the exploitation of in vitro-generated variability has become part of the routine breeding practice of many commercial enterprises.

### Disadvantages

One of the serious limitations of somaclonal variation which makes it comparatively difficult to use is that, despite the identification of factors affecting the variation response of a given plant species, it is still not possible to predict the outcome of a somaclonal program (Karp 1992) as it is random and lacks reproducibility. Further, as a large number of genetic changes are based on point mutations or chromosome rearrangements, most R_1_ segregate. Therefore for quantitative traits such as yield, it is virtually impossible to select individuals with improvements in the R_1_ generation. Though techniques for selection of somaclones resistant to various biotic and abiotic stresses had been worked out in many horticultural crops, unfortunately, no in vitro selection methods exist for complicated traits such as yield, soluble solids, sweetness, texture or shelf life (Evans 1989).

Somaclonal variation can become a part of plant breeding provided they are heritable and genetically stable. Only a limited numbers of promising varieties so far had been released using somaclonal variations. This is perhaps due to the lack of interaction between plant breeders and tissue culture scientists, and non-predictability of somaclones (Jain 2001). Further, though the new varieties have been produced by somaclonal variation, in a large number of cases improved variants have not been selected due to (1) the variations were all negative; (2) positive changes were also altered in negative ways; (3) the changes were not novel, or (4) the changes were not stable after selfing or crossing (Karp 1992).

## Recovery of somaclonal variants

The recovery of variants can be improved by promoting the factors which are responsible for the development of somaclonal variations such as protoplast culture (Kothari et al. 2010) and employing callus and cell suspension culture for several cycles and regeneration of large number of plants from long-term cultures (Barakat and El-Sammak 2011). Indirect organogenesis is an important means of retrieving genetic variation through somaclones with useful traits of agronomic or industrial use. Besides, plant genotype is a major factor, which determines the type and frequency of somaclonal variation. For instances, Solanaceous plants like potato (Sharma et al. 2007) and tomato (Bhatia et al. 2005) produce a gamut of somaclonal variation than many other commercial horticultural crops. However, to be of practical value, the frequency of somaclonal variation should be sufficient enough to select desirable traits, and the selected lines should perform well under multiple environments (Duncan 1997). The efficiency of recovering variants in vitro can further be enhanced by applying selection pressure through screening of desirable traits, e.g., in vitro selection for tolerance against abiotic and biotic stresses (Barakat and El-Sammak 2011). This attains more significance in view of the fact that the selection of desirable traits takes several years and many generations under field conditions. In vitro selection can shorten considerably the time for the selection of desirable traits under in vitro selection pressure with minimal environmental interaction, and can complement field selection (Jain 2001).

The recovery of somaclones can be increased by combining micropropagation with induced mutagenesis in vitro (Afrasiab and Iqbal 2010). Kuksova et al. (1997) noted that somaclonal variation and mutagens can be combined to increase the frequency of induced mutation. Likewise, irradiation followed by adventitious bud regeneration has been reported to have allowed the recovery of mutants with useful agronomic traits in *Gypsophila paniculata* L. (Barakat and El-Sammak 2011). Yang and Schmidt (1994) treated in vitro leaves of the cherry rootstock ‘209/1’ (*Prunus cerasus* × *P. canescens*) with X-rays with LD50 close to 20 Gy. Among plants regenerated from leaves with 20 Gy, one was phenotypically different, and was subsequently isolated and cloned. This somaclone was extremely dwarfed and was stable in both greenhouse and field tests. Employing more than one mutagen results in further improvement in recovery of somaclones in vitro. Murti et al. (2013) exposed the strawberry ‘DNKW001’ to the doses of 0, 30, 80, 130, 180, 230, 280, 300 and 325 Gy and similar doses of gamma rays + EMS 7 µM treatments. Their results showed that Gamma ray irradiation + EMS was more effective to generate more type and magnitude of variants. Purwati and Sudarsono (2007) regenerated four variant lines in abaca banana from (1) embryogenic calli; (2) ethyl methyl sulphonate (EMS)-treated embryogenic calli; (3) EMS-treated embryogenic calli, followed by in vitro selection on Foc (*Fusarium oxysporum* f.sp. cubense) culture filtrate (EMS + CF line) and (4) EMS-treated embryogenic calli, followed by in vitro selection on fusaric acid. The Foc resistance abaca variants were successfully identified from four tested abaca variant lines, although with different frequencies. However, more Foc resistance abaca plants were identified from EMS + CF line than the others. Earlier, Bidabadi et al. (2012) suggested that the subjecting of shoot tips cultures of banana to EMS (200 mM) treatments could provide an alternative strategy for inducing variants. Recently, Iuliana and Cerasela (2014) suggested irradiation of in vitro raised plants with ultraviolet radiations (UV-C) for induction of somaclones in potato.

## Application of somaclonal variations

It is well accepted that somaclonal variations arising out of unique tissue culture environment are very often noticed phenomenon in clonally propagated plants, which can advantageously be utilized as a source of new variation in horticultural crops (Karp 1995). However, suitable tools for detection, evaluation, identification and improvement of resistant clones should be designed in order to realize the benefits of such variations (Sahijram et al. 2003). Crop improvement through somaclonal variation enables breeders to obtain plants tolerant to the biotic or abiotic stress, such as drought, high salinity, high or low soil pH and disease tolerance (Yusnita et al. 2005). A number of cultivars have been developed through somaclonal variation in different horticultural crops for a range of useful traits, which are presented in Table 3.

**Table 3:** In vitro selection of desirable traits and development of some commercially exploited varieties through somaclonal variation in different horticultural crops

S. no.	Horticultural crop	Characteristic of somaclone	References
1	*Aglaonema*	Cultivar ‘Moonlight Bay’ and ‘Diamond Bay’ from ‘Silver Bay,’ and ‘Emerald Bay,’ from ‘Golden Bay’	Henny et al. (1992, 2003)
2	Apple (*Malus* × *domestica* Borkh.)	Resistance to *Erwinia amylovora*	Chevreau et al. (1998)
3	Apple rootstocks M 26 and MM 106 (*Malus pumila* Mill.)	Resistance to *Phytophthora* *cactorum*	Rosati et al. (1990)
4	Apple rootstock Malling 7	Resistance to white root rot (*Dematophora necatrix*)	Modgil et al. (2012)
5	*Anthurium* sp.	‘Orange Hot’ derived from ‘Red Hot’ clone	Henny and Chen (2011)
6	Banana (*Musa acuminata* L.)	Semi-dwarf and resistant to *Fusarium* wilt TC1-229	Tang et al. (2000)
		Larger bunch size var. TC2-425; Resistant to *Fusarium* *oxysporum* f. sp. cubense (Foc) race 4; bunch 40 % heavier than cv. Formosana	Hwang (2002)
		*Fusarium* wilt-resistant somaclonal variants of banana cv. Rasthali	Ghag et al. (2014)
		Var. CIEN-BTA-03, resistant to yellow Sigatoka	Giménez et al. (2001)
		10 somaclones; GCTCV215-1 released for commercial planting	Hwang and Ko (1992, 2004)
		Var. CUDBT-B1, reduced height and early flowering	Martin et al. (2006)
		Var. Tai-Chiao No. 5, superior horticultural traits and resistance to *Fusarium* wilt	Lee et al. (2011)
7	Begonia (*Begonia* × *elatior*)	Plant morphology, number of flowers per plant, and flower size	Jain (1997)
8	Brinjal (*Solanum melongena* L.)	Stress-tolerant somaclone selection	Ferdausi et al. (2009)
9	Blackberry	Thornless var. ‘Lincoln Logan’	Hall et al. (1986)
10	Capsicum (*Capsicum annuum* L.)	Yellow fruited var. Bell sweet	Morrison et al. (1989)
11	*Calthea roseopicta*	Developed common cultivars like Angela, Cora, Dottie, Eclipse and Saturn	Chao et al. (2005)
12	Carrot (*Daucus carota* L.)	Resistance to leaf spot (*Alternaria dauci*)	Dugdale et al. (2000)
		Resistant to drought	Rabiei et al. (2011)
13	Carnation (*Dianthus caryoplyllus* L.)	Resistant to *Fusarium oxysporum* f. sp. *dianthi*	Esmaiel et al. (2012)
14	Celery (*Apium graveolens* L.)	*Fusarium* resistant var. UC-TC	Heath-Pagliuso and Rappaport (1990)
		Multiple-resistant (insect resistance against *Spodoptera* *exigua* and disease resistance against *Fusarium* yellow) somaclones K-26, K-108 and K-128	Diawara et al. (1996)
15	*Celosia argentea* L.	Resistance to nematode	Opabode and Adebooye (2005)
16	*Cereus peruvianus*	Shoots with different areoles characteristics	Resende et al. (2010)
17	Chili pepper (*Capsicum annuum* L.)	Early flowering and increase of yield components	Hossain et al. (2003)
18	Chrysanthemum (*Dendranthema grandiflora*)	Variation in leaf, flower shape and petal size	Ahloowalia (1992)
		Daisy type chrysanthemum	Jevremović et al. (2012)
		Attractive variants with changed inflorescence colors	Miler and Zalewska (2014)
19	*Citrus* spp.	Resistant to *Phoma tracheiphila*	Deng et al. (1995)
		Salinity tolerance	Ben-Hayyim and Goffer (1989)
20	*Cuphea viscosissima* Jacq.	Significantly superior over the parents for mean plant height, leaf area, seed yield, per cent caprylic acid and lauric acid contents	Ben-Salah and Roath (1994)
21	*Cymbopogon winterianus* Jowitt	Aromatic grass var. CIMAP/Bio-13 with 50–60 % increased oil yield	Mathur et al. (1988)
		Increased total oil yield and quality with high geraniol content	Nayak et al. (2003)
	*Cymbopogon martinii*	Increased oil content	Patnaik et al. (1999)
22	*Dieffenbachia* sp.	Novel and distinct foliar variegation with taller, larger canopy and longer leaves than ‘Camouflage’ parental plants	Shen et al. (2007)
23	Garlic (*Allium* *sativum* L.)	Consistently higher bulb yield than the parental clone	Vidal et al. (1993)
		Resistance against the pathogenic fungi ‘*Sclerotium* *cepivorum’*	Zhang et al. (2012)
24	*Geranium* spp.	Vigourous and attractive flower	Skirvin and Janick (1976)
		Isomenthone-rich somaclonal mutant	Gupta et al. (2001)
		Cv. ‘CIM Pawan, a somaclone of the Bourbon type variety Bipuli, with more herbage and essential oil yield than Bipuli	Saxena et al. (2008)
25	Gerbera (*Gerbera jamesonii* Bolus)	Novel cultivars	Minerva and Kumar (2013)
26	Ginger (*Zingiber officinale* Rosc.)	Tolerant to wilt pathogen (*Fusarium oxysporum* f.sp. *zingiberi* Trujillo)	Bhardwaj et al. (2012)
27	Grapevine (*Vitis vinifera* L.)	Resistant to *Botrytis cinerea* and *Plasmopara viticola*	Kuksova et al. (1997)
28	*Haemerocallis* spp.	Dwarf, short flowers, male sterile var. Yellow Tinkerbell	Griesbach (1989)
29	*Hedychium* (ornamental ginger)	Ramata, dwarf and variegated cultivar	Sakhanokho et al. (2012)
30	*Java citronella* (*Cymbopogon winterianus*)	Somaclonal variant variety CIMAP/Bio-13, which yields 37 % more oil and 39 % more citronellon than the control variant	Mathur (2010)
31	Kiwi fruit (*Actinidia deliciosa*)	5 somaclones, derived from cv. Tamuri, tolerant to NaCl	Caboni et al. (2003)
32	Mango (*Mangifera indica* L.)	Resistant to *Colletotrichum gleosporiensis*	Litz et al. (1991)
33	Mint (*Mentha arvensis*)	Increased herb and oil yield	Kukreja et al. (1991; 2000)
34	Myrobolan (*Prunus cerasifera* Erhr)	Water logging-tolerant clone variant (S.4) of myrobolan rootstcock Mr.S 2/5 for peach cv. Sun Crest	Iacona et al. (2013)
35	Olive (*Olive europea*)	Bush olive somaclone (BOS), columnar olive somaclone (COS)	Leva et al. (2012)
36	Patchouli (*Pogostemon patchouli*)	Higher herb yield and essential oil content	Ravindra et al. (2012)
37	Pea (*Pisum sativum* L.)	Resistance to *Fusarium solani*	Horáček et al. (2013)
38	Peach (*Prunus persica* L.)	Somaclones S156 and S122 resistant to leaf spot, moderately resistant to canker in cvs. Sunhigh and Red haven	Hammerschlag and Ognjanov (1990)
		Resistant to root-knot nematode (*Meloidogyne* *incognita* Kofoid and White)	Hashmi et al. (1995)
		Somaclone S 122-1 was found resistant to bacterial canker (*Pseudomonas syringae* pv. *syringae*)	Hammerschlag (2000)
39	Pear (*Pyrus* sp.)	Resistant to *Erwinia amylovora*	Viseur (1990)
	Pear rootstock (*Pyrus communis* L.) ‘Old Home × Farmingdale (OHF 333)’	Tolerance to the fire blight	Nacheva et al. (2014)
40	*Philodendron*	Cultivars ‘Baby Hope’ from ‘Hope’	Devanand et al. (2004)
41	*Picrorhiza kurroa*	Higher glycoside contents including kutkoside and picroside I in somaclone 14-P derived through *Agrobacterium* *rhizogenes* mediated transformed hairy root cultures of *P. kurroa*	Mondal et al. (2013)
42	Pineapple (*Ananas comosus* L., Merr.)	Spineless variant	Jaya et al. (2002)
		Cvs. P3R5 and Dwarf, variation in fruit color, growth habit, fruit size and length of plant generation cycle	Pérez et al. (2009, 2012)
43	Potato (*Solanum tuberosum* L.)	Non-browning var. White Baron	Arihara et al. (1995)
		Somaclones for heat tolerance	Das et al. (2000)
		Somaclones IBP-10, IBP-27 and IBP-30, derived from cultivar Desiree, showed higher resistance to *Alternaria solani* and *Streptomyces scabiei*	Veitia-Rodriguez et al. (2002)
		Improved size, shape, appearance, starch content and starch yield	Thieme and Griess (2005)
		Superior processing attributes than cv. ‘Russet Burbank’	Nassar et al. (2011)
		High-yielding genotype SVP-53	Hoque and Morshad (2014)
		Increased phytonutrient and antioxidant components over cv. ‘Russet Burbank’	Nassar et al. (2014)
44	Quince A (*Cydonia oblonga*)	High soil pH	Dolcet-Sanjuan et al. (1992), Marino et al. (2000)
45	*Stevia rebaudiana*	High glycoside contents (steviol, stevioside, and rebaudioside)	Khan et al. (2014)
46	Strawberry (*Fragaria* sp.)	Resistant to *Fusarium oxysporum f. sp. fragariae*	Toyoda et al. (1991)
		Resistant to *Alternaria alternate*	Takahashi et al. (1993)
		Resistant to *Phytophthora cactorum*	Battistini and Rosati (1991)
		Improved horticultural traits	Biswas et al. (2009)
		Resistant to *Verticillium dahliae* Kleb	Zebrowska (2010)
		‘Serenity’, a paler skin-colored, late season, resistant to powdery mildew and *Verticillium* wilt somaclonal variant of the short-day cv. ‘Florence’	Whitehouse et al. (2014)
47	Sweet potato (*Ipomea batatas* L. Lam.)	Tolerant to salinity	Anwar et al. (2010)
48	Sweet orange (*Citrus sinensis* (L.) Osb.)	Somaclone of OLL (Orie Lee Late) sweet orange; late maturing; suitable for fresh market or processing, exceptional juice quality and flavor	Grosser et al. (2015)
49	St. Augustine grass [*Stenotaphrum* *secundatum* (Walt.) Kuntze]	Freeze-tolerant somaclonal variant SVC3	Li et al. (2010)
50	*Syngonium podophyllum* Schott	22 cultivars, derived from original ‘White Butterfly’ clone, with distinct and stable foliage characteristics	Henny and Chen (2011)
51	Tomato (*Lycopersicon esculentum* L.)	High solid contents var. DNAP9	Evans (1989)
52	Tulip (*Tulipa* sp.)	“Bs6”, selected from among the micropropagated plants of the cultivar ‘Blue Parrot’ with red-violet colored longer flower and stem	Podwyszynska et al. (2010)
53	Torenia (*Torenia fournieri*)	Flower color somaclonal variants	Nhut et al. (2013)
54	Turmeric (*Curcuma longa* L.)	High essential oil yielding somaclones	Kar et al. (2014)
		Turmeric somaclone resistant to *Fusarium oxysporum* f.sp. Zingiberi	Kuanar et al. (2014)
55	Indian ginseng (*Withania somnifera* (L.) Dunal)	Withanolide (12-deoxywithastramonolide)-rich somaclonal variant	Rana et al. (2012)

## Conclusions

Several strategies have been followed to ascertain the genetic fidelity of the in vitro produced progenies in view of the fact that the commercial viability of micropropagation technology is reliant upon maintenance of genetic fidelity in the regenerated plants. Therefore, a thorough assessment of micropropagated plants becomes very critical, especially, for perennial crops such as fruit species, which have a long pre-bearing growth period. The efficiency and sensitivity of new molecular tools has enabled us to detect somaclonal variation at an early stage. These tools have become very useful for the rapid detection and accurate identification of variants. Nevertheless, the morphological and cytological assays should continue to remain as the primary and essential assay for the sustained success of fidelity tests associated with production of clonal plants. Though, on one hand, tissue culture-induced variations pose a major threat to the genomic integrity of regenerated plants, they provide tools for improvement to plant breeders, particularly for crops with a narrow genetic base, i.e., self pollinated and vegetatively propagated. Irrespective of our goal either for production of true-to-the type planting material or creation of variability, a multidisciplinary approach (involving concerned sciences of horticulture, genetics and plant breeding, physiology, cytology and molecular biology) with all our previous knowledge and experience should be followed to achieve the desideratum.
